# Towards a Coronavirus-Based HIV Multigene Vaccine 

**DOI:** 10.1080/17402520600579168

**Published:** 2006

**Authors:** Klara K. Eriksson, Divine Makia, Reinhard Maier, Burkhard Ludewig, Volker Thiel

**Affiliations:** Research Department Kantonal Hospital Saint Gallen Saint Gallen 9007 Switzerland

## Abstract

Human immunodeficiency virus (HIV) infection represents one of the major health threats in the developing world. The costly treatment of infected individuals with multiple highly efficient anti-HIV drugs is only affordable in industrialized countries. Thus, an efficient vaccination strategy is required to prevent the further spread of the infection. The molecular biology of coronaviruses and particular features of the human coronavirus 229E (HCoV 229E) indicate that HCoV 229E-based vaccine vectors can become a new class of highly efficient vaccines. First, the receptor of HCoV 229E, human aminopeptidase N (hAPN or CD13) is expressed mainly on human dendritic cells (DCs) and macrophages indicating that targeting of HCoV 229E-based vectors to professional antigen presenting cells can be achieved by receptor-mediated transduction. Second, HCoV 229E structural genes can be replaced by multiple transcriptional units encoding various antigens. These virus-like particles (VLPs) containing HCoV 229E-based vector RNA have the ability to transduce human DCs and to mediate heterologous gene expression in these cells. Finally, coronavirus infections are associated with mainly respiratory and enteric diseases, and natural transmission of coronaviruses occurs via mucosal surfaces. In humans, HCoV 229E causes common cold by infecting the upper respiratory tract. HCoV 229E infections are mainly encountered in children and re-infection occurs frequently in adults. It is thus most likely that pre-existing immunity against HCoV 229E will not significantly impact on the vaccination efficiency if HCoV 229E-based vectors are used in humans.


*Clinical & Developmental Immunology*, June–December 2006; 13(2–4): 353–360

Taylor & Francis

Taylor & Francis Group

### Towards a coronavirus-based HIV multigene vaccine

KLARA K. ERIKSSON, DIVINE MAKIA, REINHARD MAIER, BURKHARD LUDEWIG, & VOLKER THIEL

*Research Department, Kantonal Hospital Saint Gallen, Saint Gallen 9007, Switzerland*

**Abstract**
Human immunodeficiency virus (HIV) infection represents one of the major health threats in the developing world. The costly treatment of infected individuals with multiple highly efficient anti-HIV drugs is only affordable in industrialized countries. Thus, an efficient vaccination strategy is required to prevent the further spread of the infection. The molecular biology of coronaviruses and particular features of the human coronavirus 229E (HCoV 229E) indicate that HCoV 229E-based vaccine vectors can become a new class of highly efficient vaccines. First, the receptor of HCoV 229E, human aminopeptidase N (hAPN or CD13) is expressed mainly on human dendritic cells (DCs) and macrophages indicating that targeting of HCoV 229E-based vectors to professional antigen presenting cells can be achieved by receptor-mediated transduction. Second, HCoV 229E structural genes can be replaced by multiple transcriptional units encoding various antigens. These virus-like particles (VLPs) containing HCoV 229E-based vector RNA have the ability to transduce human DCs and to mediate heterologous gene expression in these cells. Finally, coronavirus infections are associated with mainly respiratory and enteric diseases, and natural transmission of coronaviruses occurs via mucosal surfaces. In humans, HCoV 229E causes common cold by infecting the upper respiratory tract. HCoV 229E infections are mainly encountered in children and re-infection occurs frequently in adults. It is thus most likely that pre-existing immunity against HCoV 229E will not significantly impact on the vaccination efficiency if HCoV 229E-based vectors are used in humans.

**Keywords:** *AIDS, vaccination, coronavirus, HIV*

**Abbreviations:** *HIV, human immunodeficiency virus; DCs, dendritic cells; AIDS, acquired immunodeficiency syndrome; HCoV, human coronavirus; MHV, mouse hepatitis virus*

### Introduction

Prophylactic vaccines against several viral infections have been developed over the last centuries leading to the eradication of smallpox and protecting many people from diseases such as measles, rubella, mumps and polio. However, a number of diseases remain against which current vaccines are suboptimal or unavailable. Furthermore, there is growing need to develop therapeutic vaccines which may boost specific immune response to persistent viruses such as human immunodeficiency virus (HIV). The critical first step in the development of antiviral vaccines is the identification of the dominant antigens contributing to the different stages of the infection, i.e. initial replication at the site of entry, spread in the host and establishment of a persistent infection. The methodology for the identification of antigens and the characterization of immunodominant epitopes is well-established and has been further advanced by approaches from the fields of proteomics and genomics (Chakravarti et al. 2000). However, the major bottle-neck in the development of new and effective vaccines is the delivery of antigens to cellular components of the immune system that initiate protective antiviral immunity. The unmatched capacity of dendritic cells (DCs) to sample antigen at sites of pathogen entry, transport pathogens and their immunogenic components to secondary

Correspondence: V. Thiel, Research Department, Kantonal Hospital Saint Gallen, Saint Gallen 9007, Switzerland. Tel: 41 71 494 2843. Fax: 41 71 494 6321. E-mail: volker.thiel@kssg.ch

ISSN 1740-2522 print/ISSN 1740-2530 online © 2006 Taylor & Francis
DOI: 10.1080/17402520600579168

lymphoid organs and to initiate activation of T cells make them the ideal target cell for antimicrobial vaccines (Steinman and Pope 2002). The excellent capacity of DCs to prime antiviral T cell responses can be readily shown *in vitro*, i.e. few DCs can activate large numbers of virus-specific T cells in a mixed lymphocyte culture (Macatonia et al. 1989; Nonacs et al. 1992). An important prerequisite for the initiation of immune responses *in vivo* is the translocation of antigens from peripheral sites into secondary lymphoid organs (Zinkernagel et al. 1997). The high potency of DCs to induce protective antiviral immunity against the non-cytopathic lymphocytic choriomeningitis virus (LCMV) *in vivo* has been shown in studies where only 100–1000 DCs presenting a specific viral antigen have to reach secondary lymphoid organs for the induction of protective antiviral T cell responses (Ludewig et al. 1998; Ludewig et al. 2000b). DC-induced CTL responses develop rapidly and DC-immunized mice are protected against acute systemic and peripheral viral challenge (Ludewig et al. 1999). Likewise, adoptive transfer of DCs pulsed with inactivated HIV-1 into severely immunocompromised mice reconstituted with human PBL resulted in the induction of protective anti-HIV-1 responses (Lapenta et al. 2003). Moreover, vaccination of SIV-infected rhesus monkeys with a cellular DC vaccine significantly suppressed viral replication (Lu et al. 2003). A recent study in untreated HIV-1 infected individuals revealed that DC-based vaccination can elicit potent immune responses against immunodeficiency viruses in humans (Lu et al. 2004).

The efficacy of vaccines can be enhanced if optimal activation/maturation of DCs is achieved. For example, DC maturation via toll-like receptor ligands augments the activation of cytomegalovirus- and HIV-specific T cell responses *in vitro* (Lore et al. 2003). Likewise, the efficiency of various vaccine formats can be greatly enhanced if activation of DCs *in vivo* is mediated via co-delivery of immunostimulatory oligonucleotides (Sparwasser et al. 1998; Ludewig et al. 2000a) or binding to heat shock proteins (Cho et al. 2000). Incorporation of DC-activating chemokines or factors prolonging DC survival into genetic vaccines has been shown to enhance the immune response against recombinant rabies virus (Pinto et al. 2003) or HIV gp120 (Biragyn et al. 2002). A potent and effective HIV vaccine should thus directly deliver antigens to DCs and induce their activation/maturation.

### HIV infection and immunity

*Prevention of HIV infection*. The thorough knowledge of the biology of HIV that has been generated over the last two decades has paved the way for a rational vaccine design. Furthermore, the progress in the understanding of the basic immunological mechanisms underlying antigen presentation (Steinman and Pope 2002), lymphocyte trafficking and activation (Luther and Cyster 2001), and immunological memory (Kaech et al. 2002) has been instrumental for the identification of the relevant parameters that ensure the induction of protective antiviral immunity. Accordingly, an efficient HIV vaccine should induce long-lasting, broad humoral and cellular responses against the immunodominant HIV antigens. In particular, the vaccine should (i) target and activate DCs, (ii) contain the immunodominant antigens recognized by CTL and Th cells, (iii) be able to display antigenic determinants that induce broadly neutralizing antibody responses, and (iv) be applicable via mucosal surfaces.

*HIV-specific CTL and Th cell responses*. CTL responses crucially contribute to control of immunodeficiency virus infection. Broad virus-specific CTL responses can be found in peripheral blood of HIV-infected humans (Betts et al. 2001; Addo et al. 2003) and the decline of plasma viral RNA during primary HIV infection is associated with the appearance of HIV-specific CTL (Borrow et al. 1994; Koup et al. 1994). Furthermore, transient *in vivo* depletion of CD8 T cells lead to a massive increase in viral load in SIV-infected monkeys, whereas extension of the depletion for more than 28 days elicited a progressive AIDS-like syndrome (Jin et al. 1999; Schmitz et al. 1999). HIV-specific Th cells can be detected in infected individuals (Pitcher et al. 1999). It is, however, not yet clear whether these cells extert direct antiviral effects. However, the good correlation of functional CD4 T cell responses against HIV (Rosenberg et al. 1997) or SIV (McKay et al. 2003) with the clinical status strongly supports the notion that intact Th cell responses are instrumental for long-term virus control. This is most likely mediated indirectly by stimulation of virus-specific CTL. Since most patients develop T cell responses against the HIV proteins env, gag or nef (Betts et al. 2001; Addo et al. 2003), a broadly applicable vaccine should elicit immune responses (at least) against these three immmunodominant antigens.

*Broadly neutralizing antibodies*. Non-neutralizing antibodies directed against viral proteins appear early after HIV infection, whereas neutralizing antibodies appear usually rather late after primary infection (Pilgrim et al. 1997). Furthermore, sera from HIV-infected individuals usually display only weak neutralizing activity against primary isolates (Moore et al. 1995). The fact that depletion of B cells in Rhesus monkeys significantly delayed the appearance of neutralizing antibodies but did not impact on the early viral clearance (Schmitz et al. 2003) supports the notion that neutralizing antibodies do not contribute significantly during initial HIV infection. However, the presence of neutralizing antibodies may alter the clinical course of SHIV infection in macaques and prevents peripartal infection (Baba et al. 2000). Conventional vaccination approaches consistently failed to induce broadly neutralizing antibody responses (McMichael and Hanke 2003). Nevertheless, distinct monoclonal antibodies have been described that are capable of neutralizing a broad range of different HIV isolates, suggesting that such antibody responses might be induced once an adequate vaccination strategy has been developed (Moore et al. 2001). For example, altering the immunodominance pattern by using CD4-HIV envelope fusion constructs that expose normally occluded and conserved antigenic regions represents such an approach for the induction of broadly neutralizing antibodies (Fouts et al. 2003). An alternative strategy for the induction of antibodies that inhibit the infection of primary T cells with different primary HIV-1 isolates has been reported recently. This promising approach takes advantage of the highly conserved caveolin-1 binding domain of HIV-1 glycoprotein 41. Neutralization of the caveolin-1 binding site in gp41 efficiently blocks HIV-1 entry in a wide range of primary cells (Hovanessian et al. 2004).

*Mucosal vaccination*. HIV is predominantly transmitted via mucosal surfaces (Pope and Haase 2003). For example, SIV rapidly crosses the epithelial layers in the cervical mucosa and infects predominantly DCs and CD4 T cells (Spira et al. 1996). Following primary infection, the virus gains access to lymphoid organs and establishes persistent infection in CD4 T cells and macrophages. It appears that constant low-level exposure to virus (via mucosal surfaces?) is associated with resistance to HIV infection (Zhu et al. 2003). Mucosal vaccination may block transmission of intravaginally or intrarectally applied SIV (Amara et al. 2001; Belyakov et al. 2001; Veazey et al. 2003) indicating that an HIV vaccine should prevent the early stage of infection and elicit long-lasting mucosal immunity.

### Coronavirus biology and suitability as viral vectors

Although immunogenic peptides or naked nucleic acid can elicit immune responses against HIV antigens, the use of viral vectors represents a superior strategy to deliver HIV antigens and/or immunostimulatory cytokines to specific target cells. However for several reasons, many virus vector systems are still limited in their ability to induce a broad and long-lasting antiviral immune response capable to prevent HIV infection and/or to reduce viral load. Moreover, the safety of DNA-based vectors such as adeno-associated-, retro- or lenti-viruses is a matter of concern, because they can integrate into the host cell genome (Dobbelstein 2003). Recombinant adenoviruses have been studied intensively as HIV vaccine candidates mainly because they can be produced to high titers. Nevertheless, high doses of recombinant adenovirus vectors have to be applied to induce antiviral immune response, most probably because they target antigens mainly to non-lymphoid organs such as the liver (Krebs et al. 2005). In contrast to viral vectors based on DNA viruses, the use of positive-stranded RNA virus-based vectors that replicate in the cytoplasm are considered as safe vectors because it is unlikely that sequences from these vectors can integrate into the host cell genome. Moreover, the safety is well documented for vectors based on widely used vaccine strains such as poliovirus (Crotty et al. 1999) or virus-like particles (VLPs) that contain replicon RNAs devoid of structural genes (Davis et al. 2000; Harvey et al. 2003). Although some of these vectors are able to target DCs and/or to induce mucosal immunity, their cloning capacity is generally restricted and the expression of multiple HIV antigens and/or immunostimulatory cytokines is limited.

Coronaviruses display a number of features that may be advantageous to overcome these limitations and, therefore, represent promising candidate vaccine vectors. Coronaviruses are enveloped viruses that are associated mainly with respiratory and enteric diseases. For example, human coronavirus 229E infects the mucosa of the upper respiratory tract and can cause common cold. Coronavirus genomes are the largest known autonomously replicating RNAs with a size of approximately 30 kb. About two thirds of the positive-stranded genome encode the replicase gene, which is comprised of two large open reading frames (ORFs). Upon infection, translation of the genomic RNA results in the synthesis of replicase gene-encoded polyproteins that are extensively processed by viral proteinases leading to the formation of a functional replicase–transcriptase complex within the cytoplasm of the infected cell (Ziebuhr et al. 2000). A hallmark of coronavirus genome expression is their unique transcription strategy. This strategy leads to the synthesis of multiple 3′ co-terminal subgenomic mRNAs, encoding mainly structural proteins. It has been shown that the synthesis of each subgenomic mRNA involves a discontinuous step by which the so-called 3′ body sequence is fused to the genomic 5′ leader sequence (Spaan et al. 1983). The fusion of leader and body sequences during discontinuous transcription is determined, at least in part, by *cis*-acting elements, termed transcription–regulatory sequences (TRS, also referred as transcription associated sequences). These elements are located at the 5′ end of the genome and at various 3′ proximal sites corresponding to the individual transcription

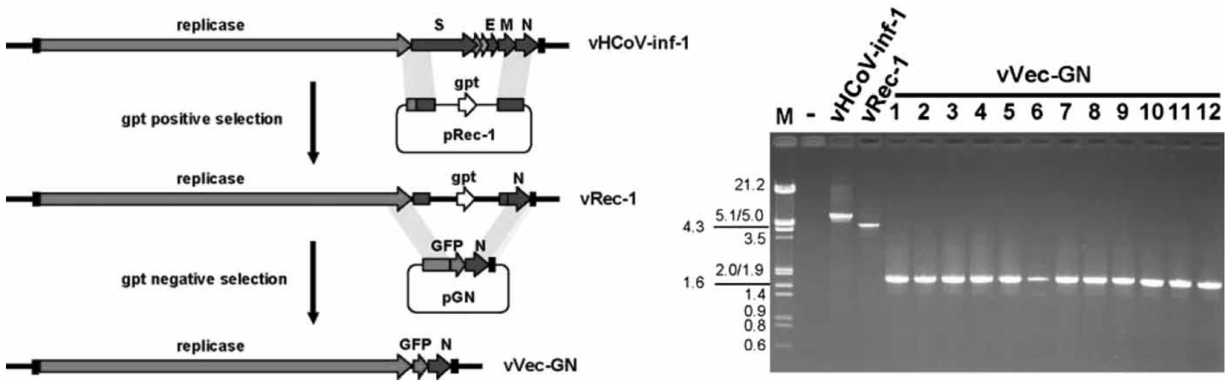

Figure 1. Mutagenesis of cloned coronavirus cDNA using vaccinia virus-mediated homologous recombination. (A) The generation of the recombinant vaccinia virus vVec-GN containing a HCoV 229E-based vector construct is illustrated. Two steps of recombination using the *E. coli* guanine phosphosribosyltransferase (gpt) as marker for positive and negative selection were performed. (B) The result of a PCR analysis from vaccinia viruses vHCoV-inf-1 (parental clone), vRec-1 (intermediate clone) and vVec-GN (desired final clone) is shown. PCR primers used in this analysis are located upstream and downstream of the region where recombination took place. Lanes 1–12 show 12 randomly picked recombinant vaccinia virus plaques obtained after gpt-negative selection, indicating the 100% recovery of desired genotypes. Notably, one vVec-GN clone was subjected to sequencing analysis of the entire coronavirus-based cDNA insert and no nucleotide changes were detected.

units. Although many studies have been performed to identify *cis*-acting sequences required for coronavirus transcription, exact borders of TRS elements have not yet been elucidated (Pasternak et al. 2001). However, short stretches of not more than 5–7 nucleotides within the TRS, called "core sequence", have been identified to determine the site of leader-body fusion of coronavirus subgenomic RNAs.

Because of the (molecular) biology of coronaviruses, coronavirus-based vectors are currently considered a promising system to genetically deliver multiple heterologous genes to specific target cells. First, coronaviruses are positive-stranded RNA viruses replicating in the cytoplasm without a DNA intermediary, making insertion of viral sequences into the host cell genome unlikely. Second, coronaviruses have the largest RNA genome known so far. Therefore, a cloning capacity of more than 6 kb is expected. Third, coronaviruses display a unique transcription process resulting in the synthesis of 6–8 subgenomic mRNAs, encoding mainly the structural genes. These genes, encoded at the 3′ third of the genome, can be replaced by multiple heterologous genes, e.g. immunogenic HIV antigens and/or immunomodulatory genes. Fourth, the receptors of human and murine coronaviruses (HCoV 229E and mouse hapatitis virus (MHV)) are expressed on human and murine DCs, respectively, indicating that efficient delivery (i.e. receptor-mediated uptake of VLPs) of heterologous genes to DCs can be achieved. Finally, the mucosal route is the natural way of coronavirus transmission.

### Establishment of a reverse genetic system for coronaviruses

We have established a reverse genetic system for coronaviruses that allows the generation of recombinant coronaviruses (Thiel et al. 2001a, 2003; Coley et al. 2005). One of the main advantages of our system is that the cloned full-length coronavirus cDNAs are amenable to site-directed mutagenesis using vaccinia virus-mediated homologous recombination. This technique is well established and has been proven to represent an efficient and precise (on the nucleotide level) method to genetically modify recombinant coronavirus cDNAs. In Figure 1, we show one example to demonstrate the ease of using vaccinia virus-mediated recombination to genetically modify coronavirus cDNA inserts.

*Generation of coronavirus-based multigene vector RNAs—transduction of human DCs.* With the reverse genetic systems available, it is now possible to make use of the unique characteristics of coronavirus transcription to develop coronavirus expression vectors. The rationale of expressing heterologous genes using coronavirus-mediated transcription is to insert a transcriptional cassette, comprised of a coronavirus TRS located upstream of the gene of interest, into a coronavirus genome, minigenome or vector RNA. We have shown for human coronavirus vector RNAs that a region of at least 5.7 kb is dispensable for discontinuous transcription (Thiel et al. 2001b). This region contained all structural genes and, therefore, our vector RNAs are not infectious. We could demonstrate that it is possible to construct a human coronavirus vector RNA capable to mediate the expression of multiple heterologous proteins. Noteworthy, this vector RNA can be packaged to VLPs if the structural proteins are expressed in trans (Thiel et al. 2003). These results indicate that coronavirus-based vector systems might be useful for heterologous gene expression, especially for longer and multiple genes.

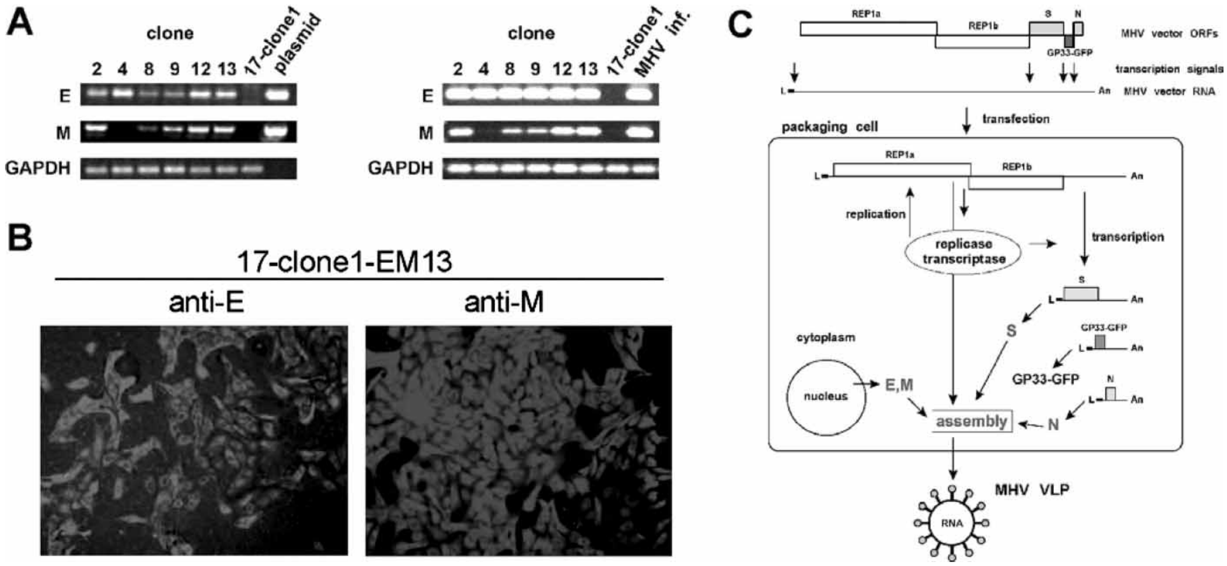

Figure 2. Packaging cell lines for the production of MHV VLPs. (A) Genomic DNA (left) or polyA-containing RNA (right) from cell clones (#2,4,8,9,12 and13) that were stably transfected with a plasmid DNA encoding the MHV *E* and *M* genes on separate transcription units were analyzed by *E* and *M* gene-specific PCR and RT–PCR, respectively. Control lanes are genomic DNA or polyA-containing RNA from parental murine 17-clone1 cells, plasmid DNA encoding E and M and polyA-containing RNA from MHV-infected 17-clone1 cells. (B) Immunofluorescence analysis of 17-clone1-EM13 cells using specific sera against MHV structural proteins E (left) and M (right). (C) Packaging strategy for the production of MHV VLPs using the MHV prototype vector MHV-Vec-A and the E- and M-expressing cell lines.

An important consideration for viral vaccine vectors is their potential for efficient delivery of their genetic material to specific target cells. For example, targeting of viral vaccine vectors to DCs is highly desirable in order to optimize vaccine efficacy. It is important to note that the HCoV 229E receptor, human aminopeptidase N (hAPN or CD13), is expressed at high levels on human DCs (Summers et al. 2001). This implies that HCoV 229E-based VLPs could be used to efficiently (receptor-mediated uptake) transduce these cells. We could demonstrate that HCoV 229E-based VLPs can be used to transduce immature and mature human DCs (Thiel et al. 2003). Therefore, this new class of safe, multigene vectors, based on HCoV 229E, represents a particularly promising tool to genetically deliver multiple antigens and immunostimulatory cytokines to human DCs.

### A reverse genetic system for mouse hepatitis virus (MHV)—establishment of a murine model to assess the efficacy of coronavirus-based vaccine vectors

In order to study the efficacy of coronavirus-based vectors *in vivo*, a small animal model is desirable. Therefore, we first established a reverse genetic system for MHV. Again we made use of vaccinia virus as cloning vector to stably propagate the full-length cDNA of MHV (strain A59). Recombinant viruses obtained from this cDNA clone were indistinguishable from the parental MHV-A59 strain in tissue culture (growth kinetics, plaque size and RNA synthesis) and in MHV-related disease models in mice (Coley et al. 2005).

With the reverse genetic system for MHV it is now possible to generate MHV-based multigene vectors that resemble their HCoV-229E counterparts. Like all coronaviruses, MHV mediates the expression of multiple subgenomic mRNAs in the infected cell. Therefore, it is possible to use the coronavirus transcription mechanism for the generation of multigene MHV vectors. Furthermore, MHV is one of the best-studied coronaviruses *in vitro* and *in vivo*. MHV grows to high titers in tissue culture ($>10^9$ pfu/ml) and the requirements for the generation of VLPs are well understood. MHV also allows for the usage of a collection of well characterized inbred and transgenic mice and a variety of established immunological techniques, indispensable for the analysis of vector-induced immune responses. Finally, it has been shown that MHV-A59 can infect murine DCs (Turner et al. 2004) and therefore, recombinant MHV vectors in the context of a murine model can serve as a paradigm for the development and evaluation of coronavirus vaccine vectors.

An important prerequisite to study the efficacy of coronavirus vaccine vectors is the availability of VLPs that can be produced to high titers. Therefore, packaging cell lines must be established which mediate the expression of coronavirus structural proteins in trans. To this end, we have generated several cell clones derived from murine 17-clone1 cells, which stably express the MHV structural proteins E and M (EM-cells). These clones have been analysed for the expression of E and M by PCR using genomic DNA as template and RT–PCR using poly(A)-containing RNA as template (Figure 2A), and immunofluorescent

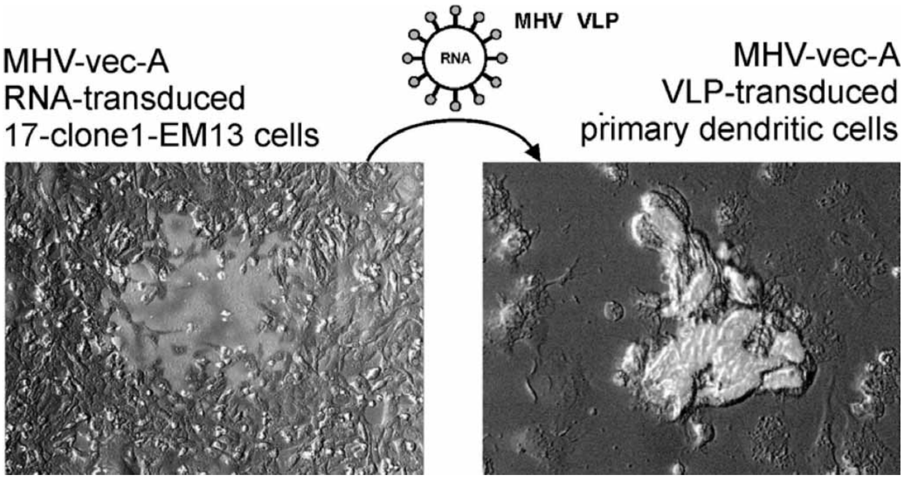

Figure 3. Generation of MHV VLPs and *in vitro* transduction of primary dendritic cells. MHV-Vec-A RNA was transfected into 17-clone1-EM13 cells by electroporation. Green fluorescence was detectable after 24 h (left). Supernatants from 17-clone1-EM13 cells were collected between 48 and 72 h following transfection and used to transduce primary DCs *in vitro*. Again, green fluorescence became apparent after 24 h (right) indicating that functional MHV VLPs have been produced in the 17-clone1-EM13 packaging cell line and that these VLPs can be used to transduce murine DCs.

microscopy (Figure 2B). Five out of six cell clones were found to contain and express both, E and M. Noteworthy, two considerations have been made before the construction of the EM cell line. First, the *E* and *M* genes in these cell lines are expressed by the cellular transcription of two separate mRNAs to minimize the possibility of reconstitution of infectious viruses by recombination of the MHV vector RNA with *E* and *M* gene mRNAs (Figure 2C). Second, in order to achieve high titer MHV VLP production, we decided to use a mouse cell line which is susceptible to MHV infection (17-clone1) for the stable transfection of *MHV E* and *M* genes. In this case, the packaging cells are susceptible to VLP-infection and we expect spread of MHV vector RNA throughout the tissue culture.

The E- and M-expressing cell lines can now be used to package MHV vector RNAs that encode (in addition to the replicase gene and the 5′ and 3′ *cis*-acting elements required for replication) the MHV structural proteins S and N (nucleocapsid protein). Therefore, we have generated a prototype MHV vector, designated MHV-Vec-A, containing the replicase gene, the 5′ and 3′ *cis*-acting elements required for replication, the structural protein S, the immunodominant CTL epitope GP33 of LCMV glycoprotein as a fusion protein with the green fluorescent protein (GP33-GFP) and the nucleocapsid protein. This vector RNA is currently being used to thoroughly assess the efficacy of VLP production in individual EM-packaging cells.

Our first experiments using MHV-Vec-A RNA for the transfection of the packaging cell line clone “17-clone1-EM13” showed that the transduction of these cells yields green fluorescent plaques indicating that our construct is functional, i.e. that the replicase complex, and the GP33-GFP fusion protein are produced. As expected, we could also observe syncytia in vector-transfected packaging cells, suggesting that a functional, cell fusion-mediating MHV S protein is present (Figure 3, left panel). Most importantly, the production of MHV VLPs is shown by the fact that transfer of supernatants from vector-transfected 17-clone1-EM13 cells to primary DC cultures leads to GFP expression in the target cells (Figure 3, right panel). Overall, these experiments provide proof-of-principle that the generation of MHV VLPs is feasible and that transgenes expressed by these replication-incompetent viruses can be targeted to DCs. We are currently in the process of testing the efficacies of MHV VLP production using the different 17-clone1-EM cell clones in order (i) to identify the best packaging cell clone; and (ii) to establish an optimized protocol for high titer VLP production.

### Conclusions

The human immunodeficiency virus (HIV) pandemic with approximately 40 million people infected worldwide and more than 4 million deaths per year, represents a major human health problem. The majority of the infections occur in Africa and HIV-induced AIDS is the leading cause of death among adults aged 15–49 years in this region. Furthermore, the numbers of infections in developing countries such as India and China have been dramatically growing over the recent years. Antiviral drug treatment has increased life expectancy and quality in western countries, but this expensive medication is usually not accessible for infected individuals in developing countries. There is thus an urgent need for an efficient and affordable vaccine.

We believe that coronaviruses have tremendous capability as tools to deliver prophylactic and therapeutic proteins to disease-relevant target cells in human. In addition, this inherently safe vector system offers the opportunity to deliver multiple proteins in combination with immunostimulatory substances. The primary goal of the outlined approach is the establishment of the coronavirus vector system and its validation in a small animal model. If this approach is feasible and effective, we should commence with the development of HCoV 229E replicon-based VLPs encoding several HIV antigens (env, gag and nef) in combination with immunostimulatory molecules. The successfully established packaging strategy will be adapted to the HCoV 229E system and should allow production of recombinant HCoV 229E VLPs. Alternatively, pseudotyped MHV-based VLPs displaying a tropism for human DCs may be used for further studies. Safety and efficacy of this vaccine preparation should be tested in an adequate non-human primate model.

### References

Addo MM, Yu XG, Rathod A, Cohen D, et al. 2003. Comprehensive epitope analysis of human immunodeficiency virus type 1 (HIV-1)-specific T-cell responses directed against the entire expressed HIV-1 genome demonstrate broadly directed responses, but no correlation to viral load. J Virol 77:2081–2092.

Amara RR, Villinger F, Altman JD, Lydy SL, et al. 2001. Control of a mucosal challenge and prevention of AIDS by a multiprotein DNA/MVA vaccine. Science 292:69–74.

Baba TW, Liska V, Hofmann-Lehmann R, Vlasak J, et al. 2000. Human neutralizing monoclonal antibodies of the IgG1 subtype protect against mucosal simian-human immunodeficiency virus infection. Nat Med 6:200–206.

Belyakov IM, Hel Z, Kelsall B, Kuznetsov VA, et al. 2001. Mucosal AIDS vaccine reduces disease and viral load in gut reservoir and blood after mucosal infection of macaques. Nat Med 7: 1320–1326.

Betts MR, Ambrozak DR, Douek DC, Bonhoeffer S, et al. 2001. Analysis of total human immunodeficiency virus (HIV)-specific CD4(+) and CD8(+) T-cell responses: Relationship to viral load in untreated HIV infection. J Virol 75:11983–11991.

Biragyn A, Belyakov IM, Chow YH, Dimitrov DS, et al. 2002. DNA vaccines encoding human immunodeficiency virus-1 glycoprotein 120 fusions with proinflammatory chemoattractants induce systemic and mucosal immune responses. Blood 100: 1153–1159.

Borrow P, Lewicki H, Hahn BH, Shaw GM, et al. 1994. Virus-specific CD8+ cytotoxic T-lymphocyte activity associated with control of viremia in primary human immunodeficiency virus type 1 infection. J Virol 68:6103–6110.

Chakravarti DN, Fiske MJ, Fletcher LD, Zagursky RJ. 2000. Application of genomics and proteomics for identification of bacterial gene products as potential vaccine candidates. Vaccine 19:601–612.

Cho BK, Palliser D, Guillen E, Wisniewski J, et al. 2000. A proposed mechanism for the induction of cytotoxic T lymphocyte production by heat shock fusion proteins. Immunity 12: 263–272.

Coley SE, Lavi E, Sawicki SG, Fu L, et al. 2005. Recombinant mouse hepatitis virus strain A59 from cloned, full-length cDNA replicates to high titers *in vitro* and is fully pathogenic *in vivo*. J Virol 79:3097–3106.

Crotty S, Lohman BL, Lu FX, Tang S, et al. 1999. Mucosal immunization of cynomolgus macaques with two serotypes of live poliovirus vectors expressing simian immunodeficiency virus antigens: Stimulation of humoral, mucosal and cellular immunity. J Virol 73:9485–9495.

Davis NL, Caley IJ, Brown KW, Betts MR, et al. 2000. Vaccination of macaques against pathogenic simian immunodeficiency virus with Venezuelan equine encephalitis virus replicon particles. J Virol 74:371–378.

Dobbelstein M. 2003. Viruses in therapy—royal road or dead end? Virus Res 92:219–221.

Fouts TR, DeVico AL, Onyabe DY, Shata MT, et al. 2003. Progress toward the development of a bacterial vaccine vector that induces high-titer long-lived broadly neutralizing antibodies against HIV-1. FEMS Immunol Med Microbiol 37:129–134.

Harvey TJ, Anraku I, Linedale R, Harrich D, et al. 2003. Kunjin virus replicon vectors for human immunodeficiency virus vaccine development. J Virol 77:7796–7803.

Hovanessian AG, Briand JP, Said EA, Svab J, et al. 2004. The caveolin-1 binding domain of HIV-1 glycoprotein gp41 is an efficient B cell epitope vaccine candidate against virus infection. Immunity 21:617–627.

Jin X, Bauer DE, Tuttleton SE, Lewin S, et al. 1999. Dramatic rise in plasma viremia after CD8(+) T cell depletion in simian immunodeficiency virus-infected macaques. J Exp Med 189:991–998.

Kaech SM, Wherry EJ, Ahmed R. 2002. Effector and memory T-cell differentiation: Implications for vaccine development. Nat Rev Immunol 2:251–262.

Koup RA, Safrit JT, Cao Y, Andrews CA, et al. 1994. Temporal association of cellular immune responses with the initial control of viremia in primary human immunodeficiency virus type 1 syndrome. J Virol 68:4650–4655.

Krebs P, Scandella E, Odermatt B, Ludewig B. 2005. Rapid functional exhaustion and deletion of CTL following immunization with recombinant adenovirus. J Immunol 174:4559–4566.

Lapenta C, Santini SM, Logozzi M, Spada M, et al. 2003. Potent immune response against HIV-1 and protection from virus challenge in hu-PBL-SCID mice immunized with inactivated virus-pulsed dendritic cells generated in the presence of IFN-alpha. J Exp Med 198:361–367.

Lore K, Betts MR, Brenchley JM, Kuruppu J, et al. 2003. Toll-like receptor ligands modulate dendritic cells to augment cytomegalovirus- and HIV-1-specific T cell responses. J Immunol 171:4320–4328.

Lu W, Arraes LC, Ferreira WT, Andrieu JM. 2004. Therapeutic dendritic-cell vaccine for chronic HIV-1 infection. Nat Med 10:1359–1365.

Lu W, Wu X, Lu Y, Guo W, et al. 2003. Therapeutic dendritic-cell vaccine for simian AIDS. Nat Med 9:27–32.

Ludewig B, Barchiesi F, Pericin M, Zinkernagel RM, et al. 2000a. *In vivo* antigen loading and activation of dendritic cells via a liposomal peptide vaccine mediates protective antiviral and anti-tumour immunity. Vaccine 19:23–32.

Ludewig B, Ehl S, Karrer U, Odermatt B, et al. 1998. Dendritic cells efficiently induce protective antiviral immunity. J Virol 72: 3812–3818.

Ludewig B, Maloy KJ, Lopez-Macias C, Odermatt B, et al. 2000b. Induction of optimal anti-viral neutralizing B cell responses by dendritic cells requires transport and release of virus particles in secondary lymphoid organs. Eur J Immunol 30:185–196.

Ludewig B, Oehen S, Barchiesi F, Schwendener RA, et al. 1999. Protective antiviral cytotoxic T cell memory is most efficiently maintained by restimulation via dendritic cells. J Immunol 163:1839–1844.

Luther SA, Cyster JG. 2001. Chemokines as regulators of T cell differentiation. Nat Immunol 2:102–107.

Macatonia SE, Taylor PM, Knight SC, Askonas BA. 1989. Primary stimulation by dendritic cells induces antiviral proliferative and cytotoxic T cell responses *in vitro*. J Exp Med 169:1255–1264.

McKay PF, Barouch DH, Schmitz JE, Veazey RS, et al. 2003. Global dysfunction of CD4 T-lymphocyte cytokine expression in simian-human immunodeficiency virus/SIV-infected monkeys is prevented by vaccination. J Virol 77:4695–4702.

McMichael AJ, Hanke T. 2003. HIV vaccines 1983–2003. Nat Med 9:874–880.

Moore JP, Cao Y, Qing L, Sattentau QJ, et al. 1995. Primary isolates of human immunodeficiency virus type 1 are relatively resistant to neutralization by monoclonal antibodies to gp120, and their neutralization is not predicted by studies with monomeric gp120. J Virol 69:101–109.

Moore JP, Parren PW, Burton DR. 2001. Genetic subtypes, humoral immunity, and human immunodeficiency virus type 1 vaccine development. J Virol 75:5721–5729.

Nonacs R, Humborg C, Tam JP, Steinman RM. 1992. Mechanisms of mouse spleen dendritic cell function in the generation of influenza-specific, cytolytic T lymphocytes. J Exp Med 176: 519–529.

Pasternak AO, van den BE, Spaan WJ, Snijder EJ. 2001. Sequence requirements for RNA strand transfer during nidovirus discontinuous subgenomic RNA synthesis. EMBO J 20: 7220–7228.

Pilgrim AK, Pantaleo G, Cohen OJ, Fink LM, et al. 1997. Neutralizing antibody responses to human immunodeficiency virus type 1 in primary infection and long-term-nonprogressive infection. J Infect Dis 176:924–932.

Pinto AR, Reyes-Sandoval A, Ertl HC. 2003. Chemokines and TRANCE as genetic adjuvants for a DNA vaccine to rabies virus. Cell Immunol 224:106–113.

Pitcher CJ, Quittner C, Peterson DM, Connors M, et al. 1999. HIV-1-specific CD4+ T cells are detectable in most individuals with active HIV-1 infection, but decline with prolonged viral suppression. Nat Med 5:518–525.

Pope M, Haase AT. 2003. Transmission, acute HIV-1 infection and the quest for strategies to prevent infection. Nat Med 9: 847–852.

Rosenberg ES, Billingsley JM, Caliendo AM, Boswell SL, et al. 1997. Vigorous HIV-1-specific CD4+ T cell responses associated with control of viremia. Science 278:1447–1450.

Schmitz JE, Kuroda MJ, Santra S, Sasseville VG, et al. 1999. Control of viremia in simian immunodeficiency virus infection by CD8(+) lymphocytes [in process citation]. Science 283: 857–860.

Schmitz JE, Kuroda MJ, Santra S, Simon MA, et al. 2003. Effect of humoral immune responses on controlling viremia during primary infection of rhesus monkeys with simian immunodeficiency virus. J Virol 77:2165–2173.

Spaan W, Delius H, Skinner M, Armstrong J, et al. 1983. Coronavirus mRNA synthesis involves fusion of non-contiguous sequences. EMBO J 2:1839–1844.

Sparwasser T, Koch ES, Vabulas RM, Heeg K, et al. 1998. Bacterial DNA and immunostimulatory CpG oligonucleotides trigger maturation and activation of murine dendritic cells. Eur J Immunol 28:2045–2054.

Spira AI, Marx PA, Patterson BK, Mahoney J, et al. 1996. Cellular targets of infection and route of viral dissemination after an intravaginal inoculation of simian immunodeficiency virus into rhesus macaques. J Exp Med 183:215–225.

Steinman RM, Pope M. 2002. Exploiting dendritic cells to improve vaccine efficacy. J Clin Invest 109:1519–1526.

Summers KL, Hock BD, McKenzie JL, Hart DN. 2001. Phenotypic characterization of five dendritic cell subsets in human tonsils. Am J Pathol 159:285–295.

Thiel V, Herold J, Schelle B, Siddell SG. 2001a. Infectious RNA transcribed *in vitro* from a cDNA copy of the human coronavirus genome cloned in vaccinia virus. J Gen Virol 82:1273–1281.

Thiel V, Herold J, Schelle B, Siddell SG. 2001b. Viral replicase gene products suffice for coronavirus discontinuous transcription. J Virol 75:6676–6681.

Thiel V, Karl N, Schelle B, Disterer P, et al. 2003. Multigene RNA vector based on coronavirus transcription. J Virol 77: 9790–9798.

Turner BC, Hemmila EM, Beauchemin N, Holmes KV. 2004. Receptor-dependent coronavirus infection of dendritic cells. J Virol 78:5486–5490.

Veazey RS, Shattock RJ, Pope M, Kirijan JC, et al. 2003. Prevention of virus transmission to macaque monkeys by a vaginally applied monoclonal antibody to HIV-1 gp120. Nat Med 9:343–346.

Zhu T, Corey L, Hwangbo Y, Lee JM, et al. 2003. Persistence of extraordinarily low levels of genetically homogeneous human immunodeficiency virus type 1 in exposed seronegative individuals. J Virol 77:6108–6116.

Ziebuhr J, Snijder EJ, Gorbalenya AE. 2000. Virus-encoded proteinases and proteolytic processing in the Nidovirales. J Gen Virol 81:853–879.

Zinkernagel RM, Ehl S, Aichele P, Oehen S, et al. 1997. Antigen localisation regulates immune responses in a dose- and time-dependent fashion: A geographical view of immune reactivity. Immunol Rev 156:199–209.

